# Long-Term Effects of Cataract Surgery on Tear Film Parameters

**DOI:** 10.1155/2013/643764

**Published:** 2013-01-20

**Authors:** Vincent D. Venincasa, Anat Galor, William Feuer, David J. Lee, Hermes Florez, Michael J. Venincasa

**Affiliations:** ^1^Department of Ophthalmology, Miami Veterans Affairs Medical Center, 1201 NW 16th Street, Miami, FL 33125, USA; ^2^Department of Ophthalmology, Bascom Palmer Eye Institute, University of Miami, 900 NW 17th Street, Miami, FL 33136, USA; ^3^Department of Epidemiology and Public Health, University of Miami, 1801 NW 9th Avenue, Miami, FL 33136, USA; ^4^Department of Endocrinology and Geriatrics, University of Miami, 1611 Northwest 12th Avenue, Miami, FL 33136, USA

## Abstract

*Purpose*. To examine the differences in tear film parameters more than 3 months postsurgery in eyes with cataract surgery (surgical eyes) versus eyes without cataract surgery (nonsurgical eyes). *Methods*. 29 patients were seen at the Miami Veterans Affairs Medical Center (VAMC) who had cataract surgery by phacoemulsification in one eye more than 3 months prior to the study date and had no history of surgical intervention in their fellow eye. Tear film parameters were measured in both eyes and compared using McNemar tests for dichotomous variables and paired and single sample *t*-tests for continuous variables. *Results*. Mean patient age was 73 (standard deviation (SD): 11); 26 patients (90%) identified themselves as White and 7 (24%) as Hispanic. The mean number of days between surgery and this study was 952 (SD: 1109). There were no statistical differences between the surgical eye and the nonsurgical eye with respect to any of the measured tear film parameters. Confidence intervals around these differences were narrow enough to exclude a substantial effect of cataract surgery. The elapsed time between cataract surgery and measurement of the tear parameters did not appear to affect the difference in parameters between the two eyes. *Conclusion*. We found that eyes that had cataract surgery more than 3 months prior to testing had no differences in their tear film parameters compared to eyes without a history of surgery.

## 1. Introduction

 Dry eye syndrome (DES) is a complex disease that presents with many symptoms, including ocular discomfort, tear film instability, and visual changes [1]. It can greatly affect patient quality of life by impairing the ability to drive, read, use a computer, and watch television among other effects [2]. Various studies have estimated the prevalence of DES to be between 5% and 34% of the population [3]. One of the major risk factors for developing novel DES or exacerbating pre-existing DES is an ocular procedure, most commonly cataract [4] or LASIK surgery [5].

Over 1.5 million cataract surgeries are performed each year in the United States alone, with an estimated annual cost of over $3.4 billion [6]. By nature of the procedure, cataract surgery typically results in some denervation of the cornea and impaired corneal sensation. Decreased corneal sensation may lead to reduced lacrimal gland tear production, which in turn can lead to dry eye symptoms [7].

With the incidence of cataracts and number of cataract surgeries performed rapidly increasing [8], it is important to study the long-term effects of this procedure. In the LASIK literature, dry eye symptoms after LASIK surgery adversely affected patient satisfaction and willingness to have the surgery again [9]. Studies have shown that cataract surgery worsens dry eye symptoms in patients with preexisting DES and induces dry eye symptoms in patients without preexisting DES in at least the first 2 months postsurgery [4, 10]. 

While the effects of cataract surgery on DES in the short term are well described [4, 10–13], there is a knowledge gap on the long-term effects of cataract surgery on dry eye symptoms and tear film parameters. This study aims to narrow the knowledge gap by examining whether or not tear film parameters become comparable in eyes with or without surgery in the long term. 

## 2. Materials and Methods

### 2.1. Study Population

Patients were prospectively recruited from the Miami VAMC clinic without regard to tear film status. Patients were excluded from the study if they were under 50 years old, had anterior segment abnormalities such as pterygium or corneal edema, used any ocular medication other than artificial tears/topical cyclosporine, or tested positive for human immunodeficiency virus (HIV), sarcoidosis, or another collagen vascular disease. To be included in our specific study, patients must have had cataract surgery by phacoemulsification with a corneal incision in one eye more than 3 months prior to data collection, and no cataract surgery in the other eye. From a collection of 263 men that were recruited to undergo tear film testing, 29 met our specific study criteria. The Miami Veterans Affairs Institutional Review Board reviewed and approved the prospective examination of patients for this study, which was conducted in accordance with the principles of the Declaration of Helsinki. Informed consent was obtained from all study participants.

### 2.2. Data Collection

We collected demographic information, past medical history, and current medical information for each patient. The ocular surface examination included tear osmolarity (TearLAB, San Diego, CA, USA, obtained once in each eye), tear breakup time (range 0–15, obtained twice and averaged in each eye), corneal staining (punctuate epithelial erosions (PEE), range 0–5) [14], Schirmer's testing with anesthesia, and morphologic and qualitative eyelid and meibomian gland information. The morphologic parameters we collected included the degree of eyelid vascularity (0-none; 1-mild engorgement; 2-moderate engorgement; 3-severe engorgement) [15] and the degree of inferior eyelid meibomian orifice plugging (0-none; 1-less than 1/3 lid involvement; 2-between 1/3 and 2/3 lid involvement; 3-greater than 2/3 lid involvement). Meibomian gland secretion (meibum) quality was rated on a scale of 0 to 4 (0-clear; 1-cloudy; 2-granular; 3-toothpaste; 4-no meibum extracted) [16]. All data was compiled into a standardized database.

### 2.3. Main Outcome Measures

The main outcomes measured were the characterization and comparison of tear film parameters in the study population in the eyes that had cataract surgery versus the eyes that had no surgery.

### 2.4. Statistical Analysis

All statistical analyses were performed with SPSS 19.0 (SPSS Inc. Chicago, IL, USA) statistical package. Descriptive statistics were used to characterize tear film parameters in our study population. McNemar tests were used to compare dichotomous variables. Paired and single sample *t*-tests were used to compare continuous variables. A *P* value of less than 0.05 was considered statistically significant. 

The difference of each tear film parameter between the two patient eyes was calculated. A 95% confidence interval (CI) was calculated for each of the differences in the tear film parameters. This interval provides, for each parameter, a likely range for the true difference between the surgical and the nonsurgical eye. To gauge whether the confidence interval for each parameter could be considered wide or narrow, we expressed it as a percentage of that parameter's range of measurements. The 1.5 × interquartile range (IQR) rule was used to check for outliers in the continuous variables. The IQR is the difference between the 25th and 75th percentile in a data set, if a value is less than the 25th percentile or greater than the 75th percentile by more than 1.5 × IQR, it is considered an outlier.

Linear regression analysis was performed to evaluate the relationship between these differences and time between the surgery and study tests. The strength of association between the difference in tear film parameters and time after surgery was summarized with linear regression (*r* ^2^). *r* ^2^ ranges from 0 to 1 and is a measure of how well the regression line approximates the real data. *r* ^2^ > 0.7 is considered a strong correlation and *r* ^2^ < 0.3 is considered a weak correlation. 

## 3. Results

### 3.1. Study Population

Twenty-nine male patients were included in the analysis. Demographic characteristics of the study population can be found in Table 1. Mean patient age was 73 (standard deviation (SD): 11). Twenty-six patients (90%) identified themselves as white and 7 patients (24%) as Hispanic. The mean number of days between surgery and study testing was 952 (SD: 1109, range: 95–3650).

### 3.2. Differences in Tear Film Parameters between Eyes

There were no statistical differences between the eye that had cataract surgery (surgical eye) and the eye without a history of surgery (nonsurgical eye) in any of tear film parameters (Table 2). There were more nonsurgical eyes with tear break-up times less than 5 compared to surgical eyes, but this was not statistically significant (*P* = 0.11, 95% confidence interval (CI), −1.77 to 1.36). Surgical eyes had a lower mean score on Schirmer's test but this difference was also not statistically significant (*P* = 0.25, 95% CI, −0.82 to 3.10). 

All of the parameters' 95% CIs were ≤12% of their respective ranges and thus narrow enough to exclude a substantial effect of cataract surgery. For all continuous variables, no outliers were present as classified by the 1.5 × IQR rule, indicating that the ranges of measurements for these variables were not excessively large due to the presence of outliers.

### 3.3. Correlation between Tear Film Parameters and Time from Surgery

No significant correlations were seen between the difference in tear film parameters for the surgical versus nonsurgical eye and time after surgery (*r* ^2^ less than 0.14 in all cases), suggesting that after 3 months tear film parameters were similar between the two eyes. 

## 4. Discussion

Prior studies have examined the effect of cataract surgery on tear film parameters and reported short-term disruptions in tear function. Ram et al. in 23 postcataract surgery patients (25 eyes) demonstrated decreased Schirmer scores and tear break-up time (TBUT) at various time points up to 2 months postoperation compared to preoperative values [12]. Li et al, in 37 postcataract surgery patients (50 eyes), similarity found decreased Schirmer and TBUT values at 1-week, 1-month, and 3-month time points compared to preoperative values [4]. These studies followed patients for a limited time period after surgery, and while there was a trend towards normalization of values, short followup limited the ability to determine whether tear film parameters returned to their baseline values. Our study's purpose, therefore, was to evaluate whether patients who had cataract surgery at least 3 months prior to testing had similar tear film parameters between the surgical and nonsurgical eyes. Indeed, our data suggests that tear film parameters recover after surgery, as all studied parameters were statistically similar between eyes. 

Ours is the first study to evaluate not only well established tests such as Schirmer's, TBUT, and corneal staining, but also other less established objective measures of tear function including tear osmolarity, eyelid vascularity, meibomian gland secretion and orifice plugging in the long term after cataract surgery. This is an important study since it can begin to lay the foundation of our knowledge on what long-term changes occur in eyes that had cataract surgery. 

One issue to consider when interpreting the results is the small sample size. In a small study, such as this one, a finding of no significant difference cannot be taken to mean that no difference exists. Therefore, we constructed 95% confidence intervals to assess the likely size of any differences between surgical and nonsurgical eyes that our significance tests might have missed. For all variables, the sizes of the confidence intervals were small compared to the ranges of the differences. Further, all continuous variables were without outliers (as classified by 1.5 × IQR), so it is unlikely that distribution of these ranges was overlarge due to skewed distributions. 

As with all studies, our conclusions must be interpreted while keeping in mind the study limitations. This study design was different than other cataract surgery studies as we did not measure tear film parameters in the same eye longitudinally. We can therefore only compare the values between eyes without commenting on whether tear film parameters “normalized” in the operated eye. Although it has been shown that fellow eyes have a substantial degree of correlation with respect to tear film parameters [17], a follow-up study with longitudinal design is needed to confirm our findings. Furthermore, our study could not evaluate the individual effects of race, age, and ethnicity on tear film function. However, by nature of its paired design, these factors were effectively controlled for, as any effect found would be independent of these demographic parameters. Finally, this study could not evaluate the impact between tear film parameters and patients' symptoms. The main source of morbidity in DES is its symptoms and prior studies have shown that correlation between symptoms and clinical tests is poor [18]. Therefore, a residual gap in knowledge is whether patients who experience increased ocular surface symptoms after surgery have an eventual decrease in their discomfort to preoperative values.

Despite these limitations, our study suggests that patients undergoing cataract surgery can be counseled that their tear film function will mirror that of the fellow eye 3 months after cataract surgery. Increasing patient understanding and giving realistic expectations often improves overall patient satisfaction and the physician-patient relationship. We hope that these findings open the door for future research to confirm our results and further study the mechanisms of tear film disruption and changes after cataract extraction, including the effect of surgery on both symptoms and corneal sensitivity, neither of which were specifically examined in this study. 

## Figures and Tables

**Table 1:** Demographic information of study population (patients who had cataract surgery in one eye and no history of surgery in the other eye). (SD: standard deviation).

Number of patients		29
Age, mean (SD) (range)		73.2 (10.7) (58–93)
Race		
White, *n* (%)		26 (90%)
Black		3 (10%)
Ethnicity		
Hispanic, *n* (%)		7 (24%)
Not Hispanic		22 (76%)
Smoking status		
Never, *n* (%)		10 (35%)
Former		14 (48%)
Current		5 (17%)
Health status		
Excellent, *n* (%)		2 (7%)
Good		19 (66%)
Fair		7 (24%)
Poor		1 (3%)
Days after surgery, mean (SD) (range)		952 (1109) (95–3650)

**Table 2:** Tear film parameters of the study population comparing non-surgical eye to surgical eye.

Tear film parameters	Nonsurgical eye (NSE)	Surgical eye (SE)	*P* value	Mean difference^I^ (SD)	95% CI^II^
Tear osmolarity, mean (SD) (range)	306.68 (13.41) (285–339)	306.21 (13.43) (284–346)	0.88	0.464 (15.58)	−5.58, 6.51
Number of patients (%)	2 (7.1%) with value >325	1 (3.6%) with value >325	1		
Corneal staining	6 (21.4%) with value >1	6 (21.4%) with value >1	1	0.071 (0.86)	−0.26, 0.40
Tear break-up time, mean (SD) (range)	8.66 (5.40) (1–15)	8.86 (4.78) (1–15)	0.79	−0.207 (4.12)	−1.77, 1.36
Number of patients (%)	14 (48.3%) with value <5	8 (27.6%) with value <5	0.11		
Schirmer's, mean (SD) (range)	13.48 (8.00) (5–30)	12.34 (7.49) (5–30)	0.25	1.138 (5.16)	−0.82, 3.10
Number of patients (%)	5 (17.2%) with value <5	3 (10.3%) with value <5	0.63		
MG quality	9 (31.0%) with value >1	6 (20.7%) with value >1	0.25	0.310 (1.07)	−0.10, 0.72
Lid vascularity	5 (17.2%) with value >1	5 (17.2%) with value >1	1	0.035 (0.33)	−0.09, 0.16
MG orifice plugging	6 (20.1%) with value >1	7 (24.1%) with value >1	1	−0.069 (0.70)	−0.34, 0.20

(^I^Mean Difference = nonsurgical eye − surgical eye; ^II^CI = confidence interval).
